# Newly developed sarcopenia after liver transplantation, determined by a fully automated 3D muscle volume estimation on abdominal CT, can predict post-transplant diabetes mellitus and poor survival outcomes

**DOI:** 10.1186/s40644-023-00593-4

**Published:** 2023-08-02

**Authors:** Sae-Jin Park, Jeong Hee Yoon, Ijin Joo, Jeong Min Lee

**Affiliations:** 1 Department of Radiology, SMG - SNU Boramae Medical Center, Seoul, Korea; 2 Department of Radiology, Seoul National University Hospital, Seoul, Korea; 3 Department of Radiology, Seoul National University College of Medicine, Seoul, Korea; 4 Institute of Radiation Medicine, Seoul National University Medical Research Center, Seoul, Korea

**Keywords:** CT, Sarcopenia, Diabetes, Deep learning, Automatic segmentation

## Abstract

**Background:**

Loss of muscle mass is the most common complication of end-stage liver disease and negatively affects outcomes for liver transplantation (LT) recipients. We aimed to determine the prognostic value of a fully automated three-dimensional (3D) muscle volume estimation using deep learning algorithms on abdominal CT in patients who underwent liver transplantation (LT).

**Methods:**

This retrospective study included 107 patients who underwent LT from 2014 to 2015. Serial CT scans, including pre-LT and 1- and 2-year follow-ups were performed. From the CT scans, deep learning-based automated body composition segmentation software was used to calculate muscle volumes in 3D. Sarcopenia was calculated by dividing average skeletal muscle area by height squared. Newly developed-(ND) sarcopenia was defined as the onset of sarcopenia 1 or 2 years after LT in patients without a history of sarcopenia before LT. Patients’ clinical characteristics, including post-transplant diabetes mellitus (PTDM) and Model for end-stage liver disease score, were compared according to the presence or absence of sarcopenia after LT. A subgroup analysis was performed in the post-LT sarcopenic group. The Kaplan–Meier method was used for overall survival (OS).

**Results:**

Patients with ND-sarcopenia had poorer OS than those who did not (*P* = 0.04, hazard ratio [HR], 3.34; 95% confidence interval [CI] 1.05 – 10.7). In the subgroup analysis for post-LT sarcopenia (*n* = 94), 34 patients (36.2%) had ND-sarcopenia. Patients with ND-sarcopenia had significantly worse OS (*P* = 0.002, HR 7.12; 95% CI 2.00 – 25.32) and higher PTDM occurrence rates (*P* = 0.02, HR 4.93; 95% CI 1.18 – 20.54) than those with sarcopenia prior to LT.

**Conclusion:**

ND-sarcopenia determined by muscle volume on abdominal CT can predict poor survival outcomes and the occurrence of PTDM for LT recipients.

**Supplementary Information:**

The online version contains supplementary material available at 10.1186/s40644-023-00593-4.

## Background

A liver transplantation (LT) has become the standard of care for patients with end-stage liver disease [1]. Among the many factors contributing to survival and outcome after LT are donor- and recipient-related variables, immunosuppressive therapy, and surgical factors. In addition to hemodynamic and metabolic disturbances, loss of muscle mass is the most common complication of end-stage liver disease [2] and negatively affects outcomes before, during, and after LT. Patients with impaired functional status as defined by sarcopenia have higher mortality rates after LT than those with a relatively preserved muscle mass [3]. In addition, pre-LT sarcopenia is associated with elevated postoperative complications and a longer hospital stay and is a predictor of mortality following LT. As such, many previous studies have investigated the effect of pre-LT sarcopenia on patient prognosis, but few studies [4, 5] have assessed the consecutive changes in muscle mass before and after LT.

Currently, as the role of imaging technology is rapidly increasing in the field of sarcopenia, there are various methods to evaluate sarcopenia using imaging [6]. Among the many imaging modalities, computed tomography (CT) is considered the best standard for investigating quantitative changes in fat and muscle [7, 8]. Although the most frequently used landmark among cross-sectional body composition studies is the L3 level of the lumbar vertebra [9], there is no standardized protocol for image acquisition of muscle mass quantification on CT. Moreover, the assessment of muscle area on a single abdominal CT image is easy and quick, but it may not be representative of the total body skeletal muscles due to regional variations of muscle volume present in a human individual [10]. Therefore, assessing sarcopenia using three-dimensional (3D) muscle volume as much as possible will be a more objective tool for diagnosing sarcopenia than using single cross-sectional muscle volume; if a fully automated technique is used using a deep learning algorithm, muscle volume will be easier to evaluate.

Herein, we investigated the effects of consecutive changes in muscle mass after LT on patient prognosis and clinical outcomes using a fully automated 3D muscle volume estimation program with a deep learning algorithm for abdominal CT.

## Methods

This retrospective study was approved by the institutional review board (IRB No. 2108–113-1245) of our institution, and the requirement for informed consent was waived.

### Patient selection

**Fig. 1:**
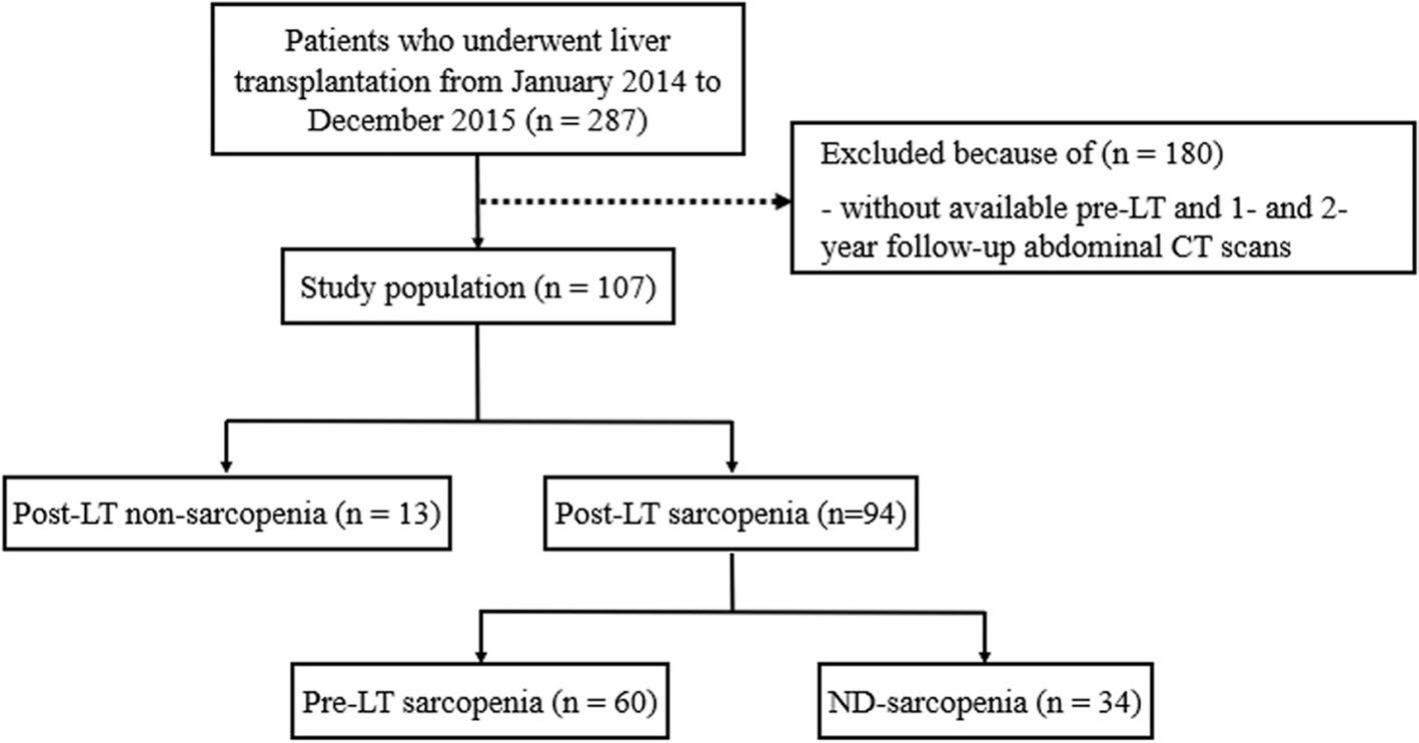
Study diagram

Among the patients who underwent LT between January 2014 and December 2015 at our institution, those with available pre-LT and 1- and 2-year follow-up abdominal CT scans were included in this study (Fig. 1).

### CT scan measurements of skeletal muscle mass

**Fig. 2:**
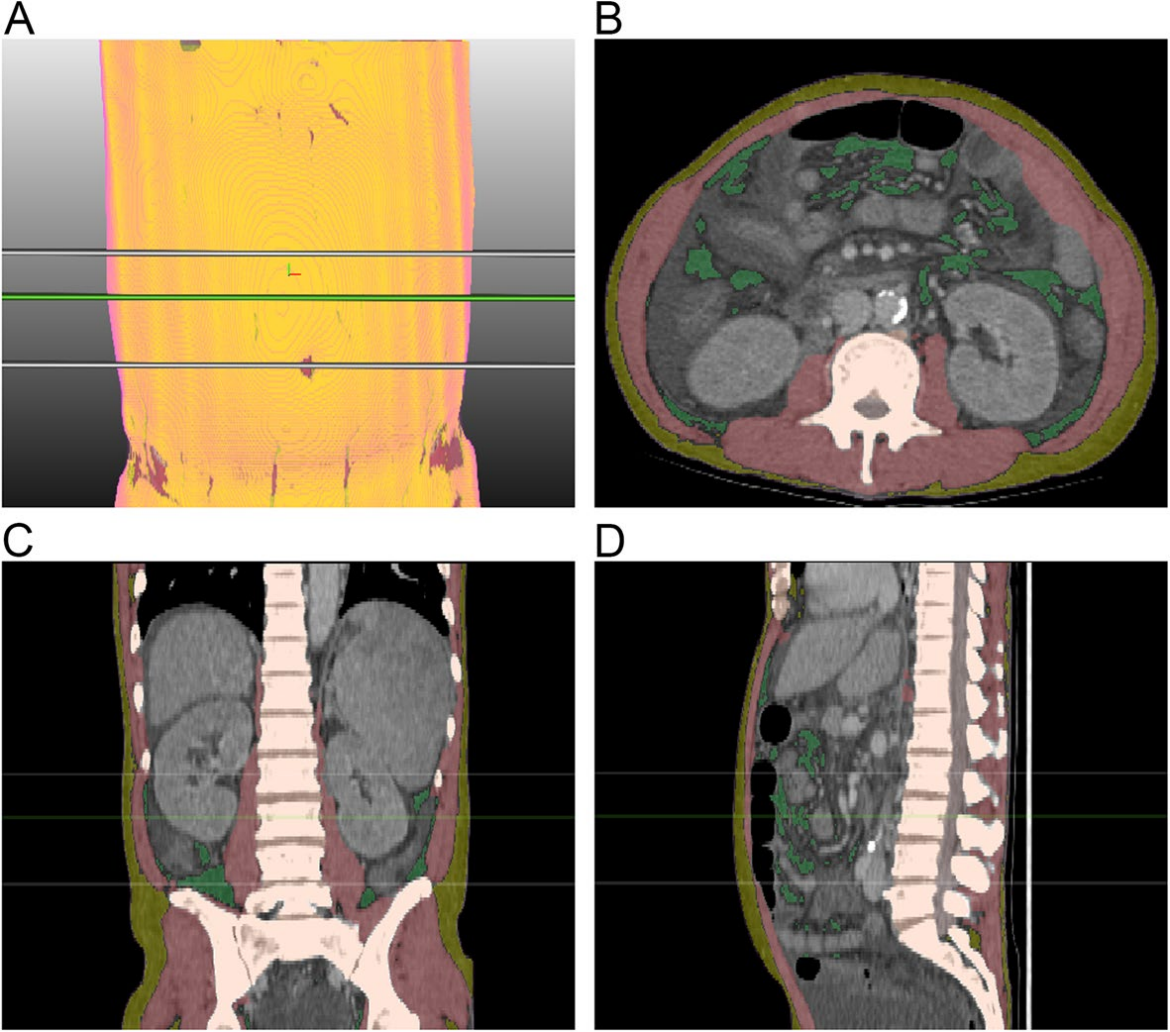
Representative three-dimensional (3D) reformatted (**A**) and cross-sectional (**B**-**D**) CT images of 3D U-Net that automatically segments CT images into a volumetric mask of seven body compartments in a 53-year-old male patient who underwent LT (body mass index, 26.2 kg/m.^2^). The overlapping lines represent the waist (white lines), L3 level (green line); skin (pink), subcutaneous fat (yellow), skeletal muscle (brown), abdominal visceral fat (light green), bone (light beige), internal organs and ascites (light gray), and central nervous system (light pink)

Abdominal CT scans were performed using various CT scanners (Additional file 1: Appendix). To measure skeletal muscle mass, unenhanced CT images of pre-LT, 1- and 2-year follow-up CT examinations were analyzed using commercially available segmentation software (MEDIP Deep Catch v1.1.4, MEDICALIP Co. Ltd., Seoul, South Korea), which allows fully automated segmentation of seven body compartments (skin, bone, skeletal muscle, visceral fat, subcutaneous fat, internal organs with vessels, and spinal cord) based on the 3D U-Net [11] (Fig. 2). The skeletal muscle area (SMA) (cm^2^) indicating the average area of skeletal muscle in the waist range was calculated by dividing the segmented muscle volume by the craniocaudal length of the automatically identified waist (from the lower margin of the last rib to the upper margin of the iliac crest) [12]. The skeletal muscle index (SMI) (cm^2^/m^2^) was calculated by dividing the average SMA by the height squared [13].

### Clinical and laboratory analysis

Clinical and laboratory data collected included sex, age, height, body weight, post-transplant diabetes mellitus (PTDM), Model for end-stage liver disease (MELD) score, hypertension (HTN), renal failure, and chronic rejection. PTDM was diagnosed according to criteria for transplant recipients were when discharged from hospital with decreased maintenance immunosuppression and in the absence of acute infection (usually 30 days); symptoms of diabetes plus random plasma glucose ≥ 200 mg/dl (11.1 mmol/L) or fasting plasma glucose ≥ 126 mg/dL (7.0 mmol/L), or 2-h glucose after a 75-g oral glucose tolerance test ≥ 200 mg/dL (11.1 mmol/L), or glycated hemoglobin ≥ 6.5% [14]. HTN was diagnosed as persistently elevated blood pressure or normal blood pressure using antihypertensive drugs after LT [15]. Chronic rejection was defined as the loss of allograft function several months to years after LT and the diagnostic criteria presented in a previous study [16]. Moreover, renal failure was defined by a serum creatinine level above 2.3 mg/dl or a glomerular filtrate rate below 50 ml/min [17]. Overall survival (OS) was calculated as the interval between LT and death or the last follow-up date.

### Definition and diagnostic criteria for sarcopenia

From the previously suggested imaging criteria for sarcopenia, we adopted the criteria of SMI < 40.8 cm^2^/m^2^ for men and SMI < 34.9 cm^2^/m^2^ for women [18]. Newly developed- (ND) sarcopenia was defined as the onset of sarcopenia 1 or 2 years after LT in patients without a history of sarcopenia before LT.

### Statistical analyses

Patients’ clinical characteristics such as sex, body mass index (BMI), SMI measured by CT, PTDM, HTN, renal failure, and chronic rejection were compared according to the presence or absence of sarcopenia after LT using the chi-square test and independent-sample t-test. OS was estimated using the Kaplan–Meier method. All statistical analyses were performed using the commercially available software (MedCalc version 19.0.3, MedCalc Software Ltd., Ostend, Belgium). *P*-values less than 0.05 indicated a statistically significant difference.

## Results

### Demographics of the study population

A total of 287 patients underwent LT during the study period. One hundred and eighty patients were excluded due to unavailable follow-up CT data (Fig. 1). As a result, 107 patients (84 males; mean age, 55.1 years) were analyzed. Demographic data details are presented in Table 1. Of the 107 patients, 71 (66.4%) had underlying hepatitis B virus, and 80 (74.8%) had undergone a living donor LT. The average BMI was 24.2 ± 3.4 kg/m^2^. PTDM occurred in 10 patients (9.3%).

**Table 1:** Demographics of study population

	Total (*n* = 107)	Post-LT non-sarcopenia (*n* = 13)	Post-LT sarcopenia (*n* = 94)	*P*-value
Sex (Male: Female)	84: 23	12: 1	72: 22	0.19
Age (years)^a^	55.1 ± 8.8	59 ± 5.1	54.6 ± 9.1	0.09
BMI, before LT (kg/m^2^) ^a^	24.2 ± 3.4	26.4 ± 3.1	23.8 ± 3.4	**0.01**
BMI, 1 year after LT (kg/m^2^)^a^	22.9 ± 3.3	26.3 ± 3.2	22.4 ± 3.1	** < 0.001**
BMI, 2 years after LT (kg/m^2^)^a^	23 ± 3.3	26.4 ± 3.1	22.5 ± 3	** < 0.001**
LDLT	80 (74.8)	9	71	0.62
Underlying malignancy	74 (69.2)	10	64	0.52
Etiology				0.17
Hepatitis B	71 (66.4)	11	60	
Hepatitis C	14 (13.1)	0	14	
Alcohol	15 (14)	2	13	
Autoimmune hepatitis	2 (1.9)	0	2	
NBNC	1 (0.9)	0	1	
Wilson disease	2 (1.9)	0	2	
Primary biliary cirrhosis	2 (1.9)	0	2	
SMI, before LT (cm^2^/m^2^)^a^	37.8 ± 8.5	47 ± 7.2	36.6 ± 7.9	** < 0.001**
SMI, 1 year after LT (cm^2^/m^2^)^a^	36 ± 8.4	45.1 ± 5.6	34.8 ± 7.9	** < 0.001**
SMI, 2 years after LT (cm^2^/m^2^)^a^	36.2 ± 7.5	45.7 ± 5.1	34.9 ± 6.8	** < 0.001**
PTDM	10 (9.3)	0	10	0.22
HTN after LT	4 (3.7)	0	4	0.45
Renal failure	9 (8.4)	1	8	0.58
Chronic rejection	1 (0.9)	0	1	0.71
MELD	17.5 ± 5.2	14.7 ± 3.1	17.9 ± 5.3	**0.04**

*BMI* Body mass index, *LDLT* Living donor liver transplantation, *NBNC* Non-B non-C, *SMI* Skeletal muscle index, *PTDM* Post-transplant diabetes mellitus, *HTN* Hypertension, *LT* Liver transplantation, *MELD* Model for end-stage liver disease

- Numbers in parentheses mean percentages

^a^mean ± SD

### SMI of pre-LT, 1- and 2-year follow ups

SMI derived from automatic segmentation of body composition on abdominal CT were 37.8 ± 8.5 cm^2^/m^2^ before LT; 36 ± 8.4 cm^2^/m^2^ 1 year after LT; and 36.2 ± 7.5 cm^2^/m^2^ 2 years after LT. Before LT, 60 patients (56.1%) were diagnosed with sarcopenia, and none showed improvement in sarcopenia after LT. Thirty-four patients were diagnosed with ND-sarcopenia (28 patients [82.4%] at 1 year after LT and 6 patients [17.6%] with additional diagnoses at 2 years after LT). Thus, 88 patients (82.2%) at 1 year after LT and 94 patients (87.9%) at 2 years after LT were diagnosed with sarcopenia.

### Comparison of outcomes between the post-LT non-sarcopenic group and post-LT sarcopenic group

Of the 107 patients, 94 were assessed as having sarcopenia on CT performed after LT, and 13 patients were not. BMI at pre-LT was 26.4 ± 3.1 kg/m^2^ in the post-LT non-sarcopenia group and 23.8 ± 3.4 kg/m^2^ in the post-LT sarcopenic group; there was a significant difference between the two groups (*P* = 0.01). There was a significant difference in BMI between the two groups in the follow-up period of 1- and 2-year follow-up periods after LT (*P* < 0.001). In the post-LT non-sarcopenic group, there was no PTDM, whereas PTDM occurred in 10 patients in the post-LT sarcopenic group: (*P* = 0.22). MELD score was statistically significantly higher in the post-LT sarcopenia group than in the post-LT non-sarcopenia group (*P* = 0.04). HTN, renal failure, and chronic rejection were not significantly different between the two groups (*P* = 0.45, *P* = 0.58, *P* = 0.71, respectively).

**Fig. 3:**
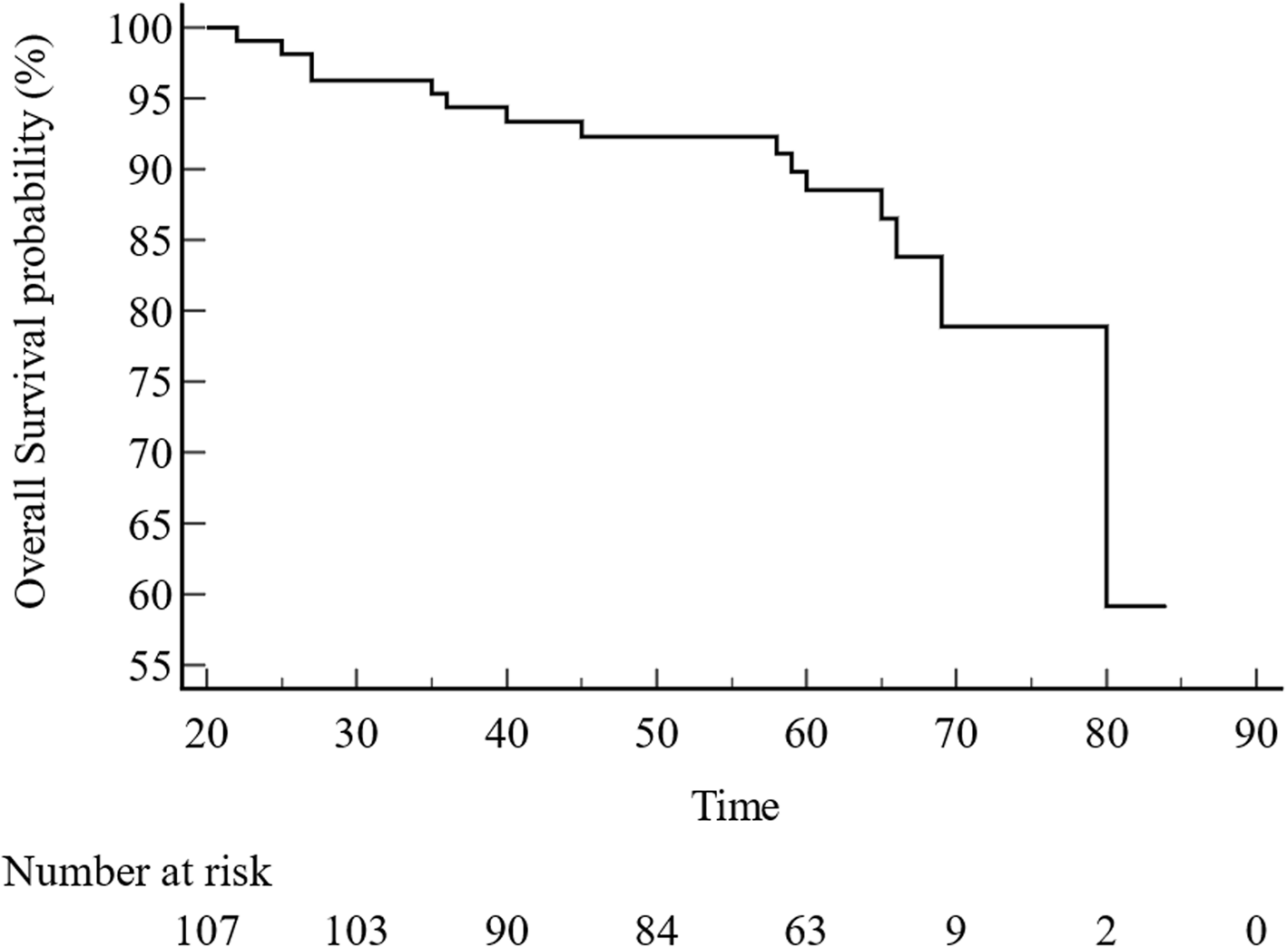
Overall survival in liver transplantation recipients

Among the 107 patients, 15 patients (14%) died during the follow-up period, one in the post-LT non-sarcopenic group and 14 in the post-LT sarcopenic group. For OS, patients with post-LT sarcopenia had poorer OS than those who did not (*P* = 0.04, hazard ratio [HR] 3.99; 95% confidence interval [CI] 1.04–15.35) (Fig. 3).

### Subgroup analyses of patients with post-LT sarcopenia

Subgroup analyses were performed for the group with pre-LT sarcopenia (*N* = 60) and the group with ND-sarcopenia after LT (*n* = 34) (Table 2). BMI at pre-LT was 22.9 ± 3.1 kg/m^2^ in the pre-LT sarcopenia group and 25.4 ± 2.9 kg/m^2^ in the ND-sarcopenic group; there was a significant difference between the two groups (*P* < 0.001). There was also a significant difference in BMI between the two groups at the 1- and 2- year follow-ups after LT (*P* = 0.01). SMI measured before LT also was significantly higher in the ND-sarcopenic group (43.7 ± 6.6 kg/m^2^) than the pre-LT sarcopenic group (32.6 ± 5.2 kg/m^2^) (*P* < 0.001). However, the SMI values measured at the 1- and 2-year follow-ups after LT were not significantly different between the two groups. Patients with ND-sarcopenia had higher PTDM occurrence rates than those with pre-LT sarcopenia (*P* = 0.02, HR 4.93; 95% CI 1.18–20.54). HTN after LT, renal failure, chronic rejection, MELD score were not significantly different between the two groups (*P* = 0.1, *P* = 0.64, *P* = 0.45, *P* = 0.08, respectively).

**Table 2:** Subgroup analysis confined to post-LT sarcopenia (*n* = 94)

	Pre-LT sarcopenia (*n* = 60)	ND-sarcopenia (*n* = 34)	*P*-value
Sex (Male: Female)	44: 16	28: 6	0.1
Age (years)^a^	53.8 ± 8.8	56 ± 9.5	0.58
BMI, before LT (kg/m^2^)^a^	22.9 ± 3.3	25.4 ± 2.9	** < 0.001**
BMI, 1 year after LT (cm^2^/m^2^)^a^	21.7 ± 3.2	23.6 ± 2.5	**0.01**
BMI, 2 years after LT (cm^2^/m^2^)^a^	21.9 ± 3.1	23.5 ± 2.5	**0.01**
LDLT	45 (75)	26 (76.5)	0.87
Underlying malignancy	42 (70)	22 (64.7)	0.6
SMI, before LT (cm^2^/m^2^)^a^	32.6 ± 5.2	43.7 ± 6.6	** < 0.001**
SMI, 1 year after LT (cm^2^/m^2^)^a^	34 ± 7.2	36.2 ± 8.9	0.2
SMI, 2 years after LT (cm^2^/m^2^)^a^	34.3 ± 7.1	35.8 ± 6.2	0.32
PTDM	3 (5)	7 (20.6)	**0.02**
HTN after LT	1 (1.7)	3 (8.8)	0.1
Renal failure	3 (5)	5 (14.7)	0.64
Chronic rejection	1 (1.7)	0	0.45
MELD	17.2 ± 4.7	19.2 ± 5.9	0.08

*BMI* Bone mass index, *LDLT* Living donor liver transplantation, *SMI* Skeletal muscle index, *PTDM* Post-transplant diabetes mellitus, *HTN* Hypertension, *LT* Liver transplantation, *ND* Newly developed, *MELD* Model for end-stage liver disease

- There was no improvement in sarcopenia in pre-LT sarcopenic group

- Numbers in parentheses mean percentages

^a^mean ± SD

**Fig. 4:**
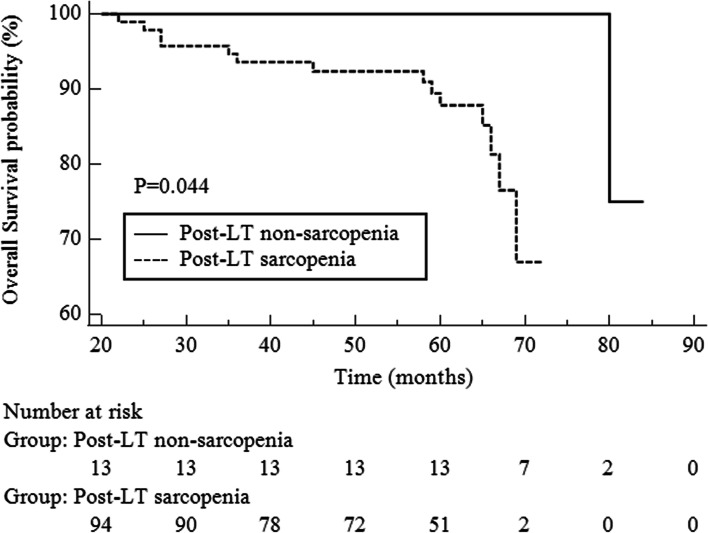
Comparison of overall survival between post-LT non-sarcopenic and post-LT sarcopenic groups

**Fig. 5:**
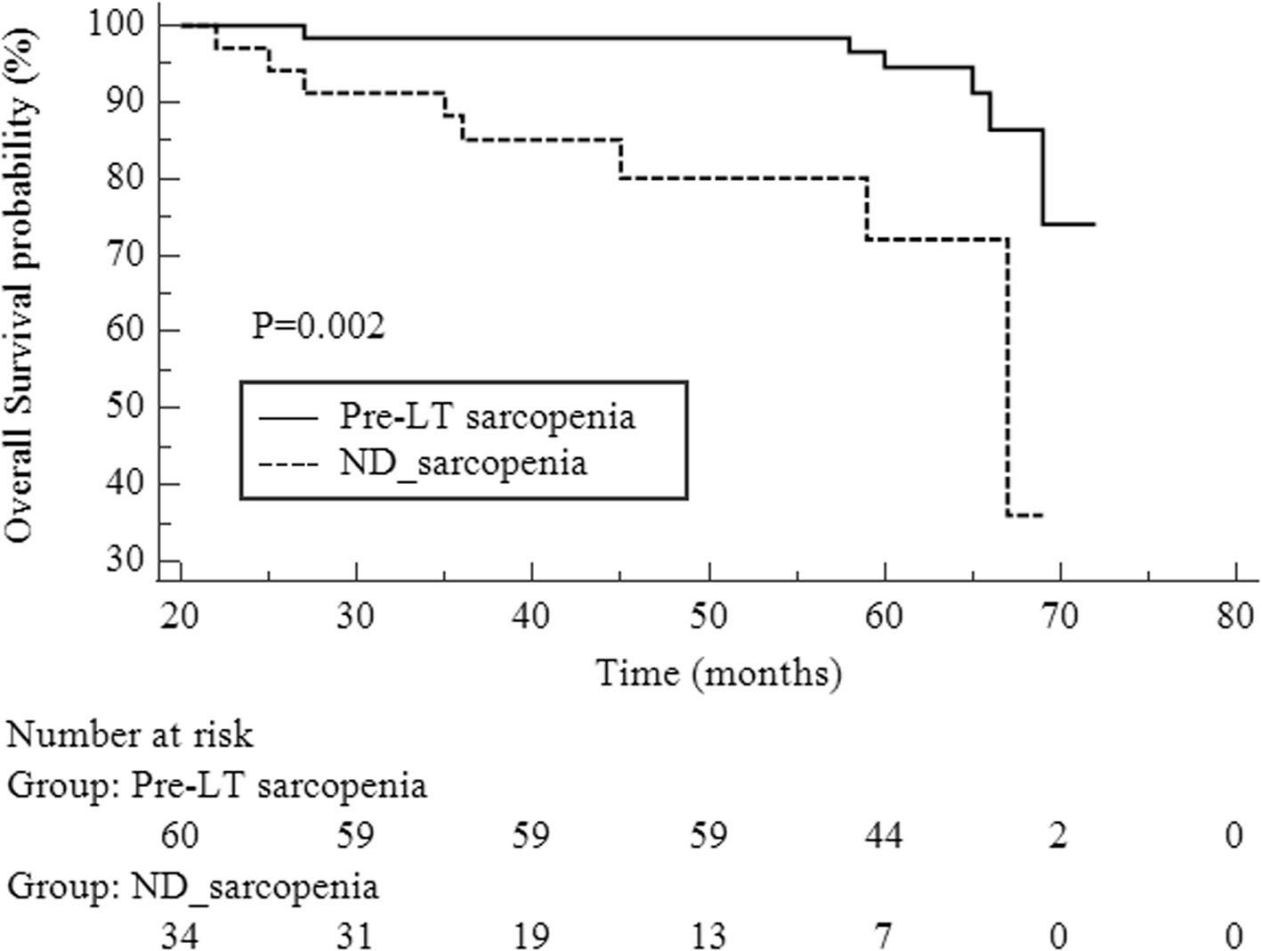
Comparison of overall survival confined to post-LT sarcopenic group

Among the 94 patients, six patients died in the pre-LT sarcopenic group and eight in the ND-sarcopenic group. For OS, the ND-sarcopenic group had significantly worse OS than the pre-LT sarcopenic group (*P* = 0.002, HR 7.12; 95% CI 2.00–25.32) (Figs. 4 and 5).

### Summary of prognostic factors for overall survival in liver transplantation recipients

Table 3 summarizes the prognostic factors for overall survival in liver transplantation recipients. Fifteen of the 107 patients (14%) died during follow-up. The estimated OS rates at 1-, 3-, and 5- years were 98.1%, 94.4%, and 88.5%, respectively. In multivariable analysis, MELD score (hazard ratio (HR) 1.15, 95% CI: 1.01–1.31, *P* = 0.03) and ND-sarcopenia (HR 3.34, 95% CI: 1.05–10.7, *P* = 0.04) were important predictors.

**Table 3:** Summary of prognostic factors for overall survival in liver transplantation recipients

	Univariable analysis		Multivariable analysis	
	HR (95% CI)	*P*	HR (95% CI)	*P*
Sex (Male: Female)	0 (1.73E-55.4E)	0.962		
Age (years)	0.98 (0.92-1.04)	0.556		
BMI, before LT (kg/m^2^)	0.93 (0.79-1.09)	0.373		
BMI, 1 year after LT (cm^2^/m^2^)	0.83 (0.71-0.98)	0.025	1.19 (0.61-2.33)	0.61
BMI, 2 years after LT (cm^2^/m^2^)	0.82 (0.69-0.97)	0.019	0.75 (0.39-1.43)	0.38
LDLT	0.86 (0.34-3.55)	0.862		
Underlying malignancy	0.99 (0.31-3.19)	0.998		
SMI, before LT (cm^2^/m^2^)	1.02 (0.97-1.09)	0.415		
SMI, 1 year after LT (cm^2^/m^2^)	0.98 (0.93-1.05)	0.627		
SMI, 2 years after LT (cm^2^/m^2^)	0.96 (0.91-1.03)	0.253		
PTDM	0.89 (0.15-9.08)	0.885		
HTN after LT	0 (1.21E-57.2E)	0.963		
Renal failure	1.46 (0.19-11.2)	0.715		
Chronic rejection	0 (3.25E-16.8E)	0.969		
MELD	1.14 (1.02-1.28)	0.023	1.15 (1.01-1.31)	**0**.**03**
ND-sarcopenia	6.51 (2.12-19.9)	0.001	3.34 (1.05-10.7)	**0**.**04**
Post-LT sarcopenia	3.99 (1.04-15.4)	0.044	0 (1.04E-5.99E)	0.95

*HR* Hazard ratio, *BMI* Bone mass index, *SMI* Skeletal muscle index, *PTDM* Post-transplant diabetes mellitus, *HTN* Hypertension, *LT* Liver transplantation, *ND* Newly developed, *MELD* Model for end-stage liver disease

## Discussion

In our study, the post-LT sarcopenia group, as determined using the fully automated 3D segmentation software, had worse OS than the group without sarcopenia. In the subgroup analysis, the occurrence of PTDM was higher in the group with ND-sarcopenia than in the group with pre-LT sarcopenia, and their OS rate was low. This indicated that if the muscle mass fell to the sarcopenia level in a group where the muscle mass was normal before LT, the prognosis was poor. Although many factors affect the prognosis of patients undergoing LT, pre-LT sarcopenia is a well-known predictive factor for poor prognosis after LT [2, 19] and sarcopenia is known to occur in between 22 and 70% of patients awaiting LT [20]. Generally, recovery from the metabolic and clinical outcomes of cirrhosis is usually achieved after a transplantation [21]. However, despite the recovery of carbohydrate, lipid, and protein metabolism in the newly functioning liver and an improved dietary intake, sarcopenia may not recover, unlike other liver-related complications, because compensatory recovery following LT generally works more strongly on fatty tissue than skeletal muscle [5]. In previous study, patients with pre-LT sarcopenia did not recover from muscle loss during the 2 years after LT [5]. Similarly, in our study, none of the patients who had sarcopenia before LT recovered from sarcopenia after LT. Based on our study results, it is necessary to manage muscle loss after LT; more specifically, since most of the ND-sarcopenia after LT occurred within 1 year (28/34, 82.4%), management of muscle mass within 1 year after LT is considered important.

PTDM is a serious metabolic complication, with an incidence rate of 10% to 36% reported for patients with LT [22–24]. This rate similar to the results of the present study (9.3%). PTDM is a disease that requires strict management, as it is known to increase the frequency of infections, cardiovascular complications, and chronic kidney disease [25, 26]. In addition to lowering the quality of life, PTDM can shorten the lifespan of patients or transplants [25, 27]. Concerning potential predictors of PTDM, it is already well known that the type or dose of immunosuppressant drugs has a significant effect on the occurrence of PTDM [28]. However, despite the development of skeletal muscle insulin resistance in diabetes and prediabetes, data on the risk of diabetes with low fat and skeletal muscle mass are limited. Tsien et al. [4] reported that muscle mass loss after post-LT is associated with the development of diabetes mellitus, but the exact mechanism is still uncertain. One hypothesis is that muscle wasting reduces the production of interleukin-15, which plays an important role in suppressing adipose tissue, reversing insulin resistance, and results in the proliferation and development of natural killer cells [29], which can lead to insulin resistance, resulting in diabetes mellitus.

In addition, there were 34 (34/47, 72.3%) patients with ND-sarcopenia after LT. Although the pathogenesis of post-transplant sarcopenia is unclear, possible mechanisms include persistent hypermetabolic hypertrophy, effects of immunosuppressive agents such as corticosteroids and calcineurin inhibitors, post-transplant infection, renal failure, and recurrence of underlying liver disease [30, 31]. As such, when sarcopenia develops after LT, the relationship between muscle loss and a patient's postoperative clinical outcome remains unknown. Although Jeon et al. [5] found that ND-sarcopenia was associated with increased mortality, the study by Tsien et al. [4] did not achieve statistical significance due to the small number of events. Further research with a large study population might be necessary to demonstrate the prognostic value of ND-sarcopenia after LT compared to pre-LT sarcopenia.

A technical note, the software using the U-net as a convolutional neural network used in this study did not measure only one level but measured the mean muscle volume at the waist through fully automated segmentation, which has proven its effectiveness in a previous study [11]. While manual or semi-automatic segmentation involves comprehensively labeling the 3D structure of each two-dimensional slice, resulting in relatively low inter-rater reliability and increased time consumption, this deep learning-based body segmentation technique allowed fully automated measurements of muscles within a few seconds, and provided high reproducibility [32]. Moreover, in contrast to manual or semi-automatic segmentation, fully automated segmentation does not require advanced knowledge after training the algorithms [33]. Currently, the recommended techniques for assessing or estimating muscle mass are dual-energy X-ray absorptiometry, CT, magnetic resonance imaging, and bio-impedance analysis [34]. Among them, CT is considered the best standard for fat and muscle quantification based on its high accuracy and reproducibility, despite its high exam costs and lack of portability [7, 8]. The high accuracy and reproducibility of CT allows high-precision mass measurements for quantifying whole-body skeletal muscle volume [35]. The widely known method for evaluating sarcopenia in CT is to measure only one level, such as L3 or L4, but it would be more accurate to include as many muscles as possible for measuring muscle mass [36, 37]. LT recipients usually have a preoperative abdominal CT scan as part of their routine evaluation and postoperative CT scans to detect complications or hepatocellular carcinoma. Therefore, the use of automated measurements of whole-body muscle volume using abdominal CT scans of LT recipients is a practical and accessible method for screening and follow-up of sarcopenia.

Our study has several limitations. First, because our study was retrospective, selection bias may have occurred. However, we tried to reduce the selection bias as much as possible by consecutively recruiting patients who underwent LT from 2014 to 2015. Second, the number of patients who were not evaluated for sarcopenia after LT was only 13, which was lower than that of the post-LT sarcopenic group. Although the survival rate in the post-LT non-sarcopenic group had a statistically significantly higher survival rate than that of the sarcopenic group, further studies with more data are needed. Third, sarcopenia was diagnosed using only muscle mass measured by CT, and muscle function or performance was not considered. In future studies, evaluation of muscle function and patient performance will be necessary. Fourth, in the presence of subcutaneous edema, X-ray attenuation in the subcutaneous fat area is increased because of the high interstitial fluid content in the adipose tissue. This can lead to difficulty in distinguishing the boundary between subcutaneous fat and abdominal muscles. In this case, manual adjustment was required (8/107, 7.5%), further refinement will be needed through these difficult cases. Nevertheless, this study is meaningful because the consecutive changes in the muscle mass of patients who received LT were assessed with 3D muscle volume estimation using automated segmentation software.

## Conclusions

ND-sarcopenia evaluated using 3D muscle volume estimation with deep learning-based automated body composition segmentation software on abdominal CT can predict the poor survival outcomes for LT recipients and the occurrence of PTDM.

## Supplementary Information

**Additional file 1.** Summaryof used CT scanners.

## Data Availability

All data generated or analyzed during this study are included in this published article. The datasets used and/or analysed during the current study available from the corresponding author on reasonable request.

## Abbreviations

LT: Liver transplantation
PTDM: Post-transplantation diabetes mellitus
SMI: Skeletal muscle index
MELD: Model for end-stage liver disease

## References

[1] Zarrinpar A, Busuttil RW (2013). Liver transplantation: past, present and future. Nat Rev Gastroenterol Hepatol.

[2] Meeks AC, Madill J (2017). Sarcopenia in liver transplantation: a review. Clin Nutr ESPEN.

[3] Selberg O, Böttcher J, Tusch G, Pichlmayr R, Henkel E, Müller MJ (1997). Identification of high- and low-risk patients before liver transplantation: a prospective cohort study of nutritional and metabolic parameters in 150 patients. Hepatology.

[4] Tsien C, Garber A, Narayanan A, Shah SN, Barnes D, Eghtesad B, Fung J, McCullough AJ, Dasarathy S (2014). Post-liver transplantation sarcopenia in cirrhosis: a prospective evaluation. J Gastroenterol Hepatol.

[5] Jeon JY, Wang HJ, Ock SY, Xu W, Lee JD, Lee JH, Kim HJ, Kim DJ, Lee KW, Han SJ (2015). Newly developed sarcopenia as a prognostic factor for survival in patients who underwent liver transplantation. PLoS One.

[6] Lee K, Shin Y, Huh J, Sung YS, Lee IS, Yoon KH, Kim KW (2019). Recent issues on body composition imaging for sarcopenia evaluation. Korean J Radiol.

[7] Cooper C, Fielding R, Visser M, van Loon LJ, Rolland Y, Orwoll E, Reid K, Boonen S, Dere W, Epstein S (2013). Tools in the assessment of sarcopenia. Calcif Tissue Int.

[8] Cruz-Jentoft AJ, Baeyens JP, Bauer JM, Boirie Y, Cederholm T, Landi F, Martin FC, Michel JP, Rolland Y, Schneider SM (2010). Sarcopenia: European consensus on definition and diagnosis: report of the European working group on sarcopenia in older people. Age Ageing.

[9] Morrell GR, Ikizler TA, Chen X, Heilbrun ME, Wei G, Boucher R, Beddhu S (2016). Psoas muscle cross-sectional area as a measure of whole-body lean muscle mass in maintenance hemodialysis patients. J Ren Nutr.

[10] Rollins KE, Gopinath A, Awwad A, Macdonald IA, Lobo DN (2020). Computed tomography-based psoas skeletal muscle area and radiodensity are poor sentinels for whole L3 skeletal muscle values. Clin Nutr.

[11] Lee YS, Hong N, Witanto JN, Choi YR, Park J, Decazes P, Eude F, Kim CO, Chang Kim H, Goo JM (2021). Deep neural network for automatic volumetric segmentation of whole-body CT images for body composition assessment. Clin Nutr.

[12] Joo I, Kwak MS, Park DH, Yoon SH (2021). Fully automated waist circumference measurement on abdominal CT: comparison with manual measurements and potential value for identifying overweight and obesity as an adjunct output of CT scan. PLoS One.

[13] Lee J, Chang CL, Lin JB, Wu MH, Sun FJ, Jan YT, Hsu SM, Chen YJ (2018). Skeletal muscle loss is an imaging biomarker of outcome after definitive chemoradiotherapy for locally advanced cervical cancer. Clin Cancer Res.

[14] Sharif A, Hecking M, de Vries AP, Porrini E, Hornum M, Rasoul-Rockenschaub S, Berlakovich G, Krebs M, Kautzky-Willer A, Schernthaner G (2014). Proceedings from an international consensus meeting on posttransplantation diabetes mellitus: recommendations and future directions. Am J Transplant.

[15] Unger T, Borghi C, Charchar F, Khan NA, Poulter NR, Prabhakaran D, Ramirez A, Schlaich M, Stergiou GS, Tomaszewski M (2020). 2020 International society of hypertension global hypertension practice guidelines. J Hypertens.

[16] Choudhary NS, Saigal S, Bansal RK, Saraf N, Gautam D, Soin AS (2017). Acute and chronic rejection after liver transplantation: what a clinician needs to know. J Clin Exp Hepatol.

[17] Section 2: AKI Definition. Kidney Int Suppl (2011). 2012;2(1):19–36. 10.1038/kisup.2011.32 PMC4089595 25018918

[18] Zhuang CL, Huang DD, Pang WY, Zhou CJ, Wang SL, Lou N, Ma LL, Yu Z, Shen X (2016). Sarcopenia is an independent predictor of severe postoperative complications and long-term survival after radical gastrectomy for gastric cancer: analysis from a large-scale cohort. Medicine (Baltimore).

[19] Kalafateli M, Mantzoukis K, Choi Yau Y, Mohammad AO, Arora S, Rodrigues S, de Vos M, Papadimitriou K, Thorburn D, O'Beirne J (2017). Malnutrition and sarcopenia predict post-liver transplantation outcomes independently of the Model for End-stage Liver Disease score. J Cachexia Sarcopenia Muscle.

[20] Kumar V, Benjamin J, Shasthry V, Subramanya Bharathy KG, Sinha PK, Kumar G, Pamecha V (2020). Sarcopenia in cirrhosis: fallout on liver transplantation. J Clin Exp Hepatol.

[21] Duffy JP, Kao K, Ko CY, Farmer DG, McDiarmid SV, Hong JC, Venick RS, Feist S, Goldstein L, Saab S (2010). Long-term patient outcome and quality of life after liver transplantation: analysis of 20-year survivors. Ann Surg.

[22] Carey EJ, Aqel BA, Byrne TJ, Douglas DD, Rakela J, Vargas HE, Moss AA, Mulligan DC, Reddy KS, Chakkera HA (2012). Pretransplant fasting glucose predicts new-onset diabetes after liver transplantation. J Transplant.

[23] Kuo HT, Sampaio MS, Ye X, Reddy P, Martin P, Bunnapradist S (2010). Risk factors for new-onset diabetes mellitus in adult liver transplant recipients, an analysis of the Organ Procurement and Transplant Network/United Network for Organ Sharing database. Transplantation.

[24] Saliba F, Lakehal M, Pageaux GP, Roche B, Vanlemmens C, Duvoux C, Dumortier J, Salamé E, Calmus Y, Maugendre D (2007). Risk factors for new-onset diabetes mellitus following liver transplantation and impact of hepatitis C infection : an observational multicenter study. Liver Transpl.

[25] Benhamou PY, Penfornis A (2002). Natural history, prognosis, and management of transplantation-induced diabetes mellitus. Diabetes Metab.

[26] de Boccardo G, Kim JY, Schiano TD, Maurette R, Gagliardi R, Murphy B, Emre S, Akalin E (2008). The burden of chronic kidney disease in long-term liver transplant recipients. Transplant Proc.

[27] Dopazo C, Bilbao I, Castells LL, Sapisochin G, Moreiras C, Campos-Varela I, Echeverri J, Caralt M, Lázaro JL, Charco R (2015). Analysis of adult 20-year survivors after liver transplantation. Hepatol Int.

[28] Montori VM, Basu A, Erwin PJ, Velosa JA, Gabriel SE, Kudva YC (2002). Posttransplantation diabetes: a systematic review of the literature. Diabetes Care.

[29] Mishra A, Sullivan L, Caligiuri MA (2014). Molecular pathways: interleukin-15 signaling in health and in cancer. Clin Cancer Res.

[30] Dasarathy S (2013). Posttransplant sarcopenia: an underrecognized early consequence of liver transplantation. Dig Dis Sci.

[31] Ma K, Mallidis C, Bhasin S, Mahabadi V, Artaza J, Gonzalez-Cadavid N, Arias J, Salehian B (2003). Glucocorticoid-induced skeletal muscle atrophy is associated with upregulation of myostatin gene expression. Am J Physiol Endocrinol Metab.

[32] Amarasinghe KC, Lopes J, Beraldo J, Kiss N, Bucknell N, Everitt S, Jackson P, Litchfield C, Denehy L, Blyth BJ (2021). A deep learning model to automate skeletal muscle area measurement on computed tomography images. Front Oncol.

[33] Toulkeridou E, Gutierrez CE, Baum D, Doya K, Economo EP. Automated segmentation of insect anatomy from micro-CT images using deep learning. bioRxiv. 2021. 10.1101/2021.05.29.446283.

[34] Walowski CO, Braun W, Maisch MJ, Jensen B, Peine S, Norman K, Muller MJ, Bosy-Westphal A (2020). Reference values for skeletal muscle mass - current concepts and methodological considerations. Nutrients.

[35] Mourtzakis M, Prado CM, Lieffers JR, Reiman T, McCargar LJ, Baracos VE (2008). A practical and precise approach to quantification of body composition in cancer patients using computed tomography images acquired during routine care. Appl Physiol Nutr Metab.

[36] Mitsiopoulos N, Baumgartner RN, Heymsfield SB, Lyons W, Gallagher D, Ross R (1998). Cadaver validation of skeletal muscle measurement by magnetic resonance imaging and computerized tomography. J Appl Physiol (1985).

[37] Prado CM, Heymsfield SB (2014). Lean tissue imaging: a new era for nutritional assessment and intervention. JPEN J Parenter Enteral Nutr.

