# Supplementary material for: Newly developed sarcopenia after liver transplantation, determined by a fully automated 3D muscle volume estimation on abdominal CT, can predict post-transplant diabetes mellitus and poor survival outcomes
Source: Cancer Imaging. 2023 Aug 2;23:73. doi: 10.1186/s40644-023-00593-4 (PMC10394977; doi:10.1186/s40644-023-00593-4)
Supplement: Supplementary file 1 — Additional file 1. Summaryof used CT scanners. [file 40644_2023_593_MOESM1_ESM.docx]

**<Appendix> Summary of used CT scanners**

| 320-channel scanners | Aquilion ONE (Canon Medical Systems) |
| --- | --- |
| 256-channel scanners | iCT 256 (Philips Healthcare) |
| 192-channel scanners | SOMATOM Force (Siemens Healthcare) |
| 128-channel scanners | IQon-spectral CT (Philips Healthcare) |
|  | Ingenuity CT (Philips Healthcare) |
| 64-channel scanners | Brilliance 64 (Philips Healthcare) |
|  | SOMATOM Definition (Siemens Healthcare) |
|  | Discovery CT750 HD (GE Healthcare) |
